# Incidence and Predictors of Advanced Liver Fibrosis by a Validated Serum Biomarker in Liver Transplant Recipients

**DOI:** 10.1155/2017/4381864

**Published:** 2017-03-19

**Authors:** Mamatha Bhat, Kathleen C. Rollet-Kurhajec, Aparna Bhat, Amanda Farag, Marc Deschenes, Philip Wong, Peter Ghali, Giada Sebastiani

**Affiliations:** ^1^Division of Gastroenterology and Hepatology, Department of Medicine, McGill University Health Centre, Montreal, QC, Canada; ^2^Division of Gastroenterology and Hepatology, Department of Medicine, University Health Network and University of Toronto, Toronto, ON, Canada; ^3^Chronic Viral Illness Service, Department of Medicine, McGill University Health Centre, Montreal, QC, Canada

## Abstract

*Background and Aims*. Serum fibrosis biomarkers have shown good accuracy in the liver transplant (LT) population. We employed a simple serum biomarker to elucidate incidence and predictors of advanced fibrosis after LT over a long follow-up period.* Methods*. We included 440 consecutive patients who underwent LT between 1991 and 2013. Advanced liver fibrosis was defined as FIB-4 > 3.25 beyond 12 months after LT.* Results*. Over 2030.5 person-years (PY) of follow-up, 189 (43%) developed FIB-4 > 3.25, accounting for an incidence of 9.3/100 PY (95% confidence interval [CI], 8.1–10.7). Advanced fibrosis was predicted by chronic HCV infection (adjusted hazard ratio (aHR) = 3.96, 95% CI 2.92–5.36, *p* < 0.001), hypoalbuminemia (aHR = 2.31, 95% CI 1.72–3.09; *p* < 0.001), and hyponatremia (aHR = 1.48, 95% CI 1.09–2.01; *p* = 0.01). LT recipients with more than 1 predictor had a higher incidence of advanced fibrosis, the highest being when all 3 predictors coexisted (log-rank: *p* < 0.001).* Conclusions*. Chronic HCV infection, hypoalbuminemia, and hyponatremia predict progression to advanced liver fibrosis following LT. Patients with these risk factors should be serially monitored using noninvasive fibrosis biomarkers and prioritized for interventions.

## 1. Introduction

Development of liver fibrosis following liver transplantation (LT) is clinically significant, given that it portends worse long-term graft and patient survival as well as potential need for retransplantation [1, 2]. Hepatic fibrosis may occur as a manifestation of recurrent or de novo chronic liver disease following LT. The incidence of hepatic fibrosis has been well defined in the LT population with hepatitis C (HCV), in whom 20–30% develop advanced fibrosis by 5 years after LT [3]. It is well established that concomitant infection with cytomegalovirus (CMV) contributes to development of fibrosis in the HCV-positive LT patient population [4]. Although the majority of those with hepatic fibrosis after LT are HCV-positive, the incidence of fibrosis in the whole population of LT recipients has not been well defined. Posttransplant metabolic syndrome commonly occurs after LT due to immunosuppressant medications and put LT recipients at risk for recurrent or de novo nonalcoholic fatty liver disease (NAFLD) [5, 6]. Predictors of hepatic fibrosis progression donor age greater than 50 years have been shown to hasten fibrosis progression, with median progression rate of 2.7 units/year and only 2.2 years till development of cirrhosis [7].

Liver biopsy has long been the gold standard to diagnose hepatic fibrosis in LT recipients. The frequency of recurrent hepatitis C and recurrent or de novo NAFLD has led some LT programs to institute annual protocol liver biopsies [8]. However, liver biopsy is impractical as a screening tool and for serial monitoring because of its invasiveness, cost, and potential for sampling error [9, 10]. Noninvasive tools have been proposed to establish the presence of advanced liver fibrosis, including the validated serum biomarker FIB-4 (based on age, transaminases, and platelets) and measurement of liver stiffness by transient elastography (Fibroscan) [10–12]. Although the FIB-4 does not enable distinction between stages of fibrosis [13], it was chosen as it incorporates an additional parameter beyond the APRI and is easily calculable. The accuracy of this score has been validated in 2 retrospective [13, 14] and 1 prospective [15] studies of fibrosis development among liver transplant recipients with all etiologies of liver disease, with an AUC ranging from 0.66 to 0.78 for fibrosis ≥ F2 by METAVIR.

The aim of this study was to determine incidence and predictors of advanced liver fibrosis after LT in a large cohort of Canadian LT recipients over a long follow-up period using a validated serum biomarker. We also aimed at developing a model based on identified predictors able to discriminate patients at higher risk of developing advanced fibrosis, on whom to target resources and interventions. Finally, we aimed at validating FIB-4 against liver biopsy in a subgroup of patients with available liver biopsy in our LT recipient population.

## 2. Patients and Methods

### 2.1. Study Design and Population

This was a retrospective study conducted at a single site, the Division of Gastroenterology and Hepatology of McGill University Health Centre, and included all eligible LT recipients. Since 1990, a computerized database on all LT recipients has been maintained into which demographic data, clinical diagnosis, laboratory results, and prescription information have been prospectively entered. In order to be included, patients had to fulfill the following criteria: (1) age > 18 years; (2) graft survival > 6 months; (3) availability of relevant serum parameters for the calculation of the serum fibrosis biomarker FIB-4 at 6 months after LT; (4) a minimum follow-up of 1 year. If patients had been retransplanted, the most recent transplant was included. Exclusion criteria were (1) FIB-4 > 3.25 at 6 months from LT; (2) no follow-up visit; (3) diagnosis of fibrosing cholestatic hepatitis.

The immunosuppressive regimen used as a standard by our liver transplant program is induction with antithymocyte globulin (ATG) and started on tacrolimus and mycophenolate mofetil (MMF) as maintenance immunosuppression and rapid prednisone taper. This is the regimen applied to non-HCV patients, and MMF is usually discontinued by 6 months post-LT. The HCV patient population is started routinely on cyclosporine and MMF as maintenance immunosuppression. We did not have data available on changes in immunosuppression (switching from cyclosporine to tacrolimus or sirolimus) during long-term follow-up.

The Institutional Research Ethic Board of the Research Institute of McGill University Health Centre approved the study, which was conducted according to the Declaration of Helsinki.

### 2.2. Outcome Measures and Serum Fibrosis Biomarker

The outcome measure was advanced liver fibrosis histologically defined as stages 3-4 by METAVIR staging system [16, 17]. Advanced liver fibrosis is a clinically meaningful threshold, because it is the hallmark of a progressive liver disease that will rapidly lead to cirrhosis [18]. FIB-4 was used to diagnose advanced liver fibrosis and was calculated as age (years) × AST/platelet count (10^9^/L) × ALT^1/2^ [11]. The outcome was defined as the second consecutive event diagnosed by standard cut-off value of FIB-4 > 3.25 for advanced liver fibrosis.

### 2.3. Clinical and Biological Parameters

Clinical parameters included age, gender, ethnicity, body mass index (BMI), primary indication for LT, Model for End-Stage Liver Disease (MELD) score at LT, date of LT, cold ischemia time, history of diabetes or hypertension, and immunosuppression used after LT. Biological parameters included platelets, AST, ALT, albumin, sodium, cholesterol, CMV, and hepatitis C status. Obesity was defined as BMI ≥ 30 kg/m^2^ [19]. Hyponatremia was defined as sodium ≤ 135 mmol/L.

### 2.4. Histological Assessment

Liver biopsy was available for a subset of patients, and it had been obtained using the Menghini technique with a 1.6 mm needle. The mean length of the liver specimen was 1.7 ± 0.4 cm. All liver biopsies were interpreted by two experienced liver pathologists. The stage of fibrosis was reported according to the Brunt classification for NASH and alcoholic hepatitis, whereas METAVIR was used for all other types of liver disease, mainly viral [16, 17]. Briefly, fibrosis was staged as follows: stage 0: no fibrosis; stage 1: portal fibrosis without septa; stage 2: portal fibrosis with few septa; stage 3: numerous septa without cirrhosis; stage 4: cirrhosis. Advanced liver fibrosis was defined as a METAVIR or Brunt score ≥ F3. Only liver biopsy specimens longer than 1 cm were included in the analysis.

### 2.5. Follow-Up

Patients were followed up until February 2014 or were censored when they either died, developed the outcome, or were at their last clinic visit. During this period, patients were followed up at varied intervals, ranging from 3 to 12 months. At each visit, complete medical history and physical examination were performed along with routine laboratory workup. Standard diagnostic and therapeutic management following LT was offered during the follow-up.

### 2.6. Statistical Analysis

Baseline (time zero) corresponded to the first visit 6 months after transplant when relevant serum parameters were available simultaneously for the calculation of the serum fibrosis biomarker FIB-4. Continuous variables were expressed as mean (standard deviation) or median (interquartile range, IQR), and categorical variables were presented as numbers (%). We compared characteristics of participants by outcome status using Student's *t*-test for continuous variables and Pearson's *χ*^2^ or Fisher's exact test for categorical variables. All tests were two-tailed and with a significance level of *α* = 0.05. We estimated incidence rates of advanced liver fibrosis (FIB-4 > 3.25) by dividing the number of participants developing the outcome by the number of person-years (PY) of follow-up. Poisson count models were used to calculate confidence intervals (CI) for incidence rates. Multivariate time-dependent Cox regression models were constructed to assess predictors of development of advanced liver fibrosis and included covariates that were determined a priori to be clinically important. The final model was adjusted for diabetes, MELD, hyponatremia, hypoalbuminemia, cyclosporine use, CMV positive recipient, and chronic HCV infection. Robust variance estimation was used in all Cox regression analyses to account for the correlation of data contributed by the same participant at multiple visits. We considered an association with the outcome significant when the 95% confidence interval (CI) excluded one. Statistical analyses were performed using R program for Windows Release 2.13.1.

## 3. Results

**Figure 1:**
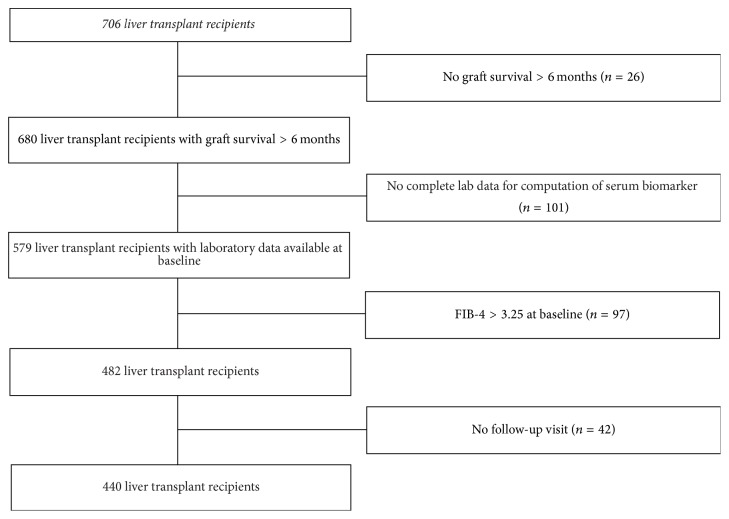
Flow chart displaying selection of study participants for analysis. Out of 706 liver transplant recipients present in the Solid Organ Transplant Unit database, 26 patients were excluded because of graft survival < 6 months and 101 patients were excluded because of missing data. After further exclusion of 97 patients who had the outcome at baseline and 42 cases without a follow-up visit, the remaining sample of 440 consecutive liver transplant recipients was included in the present study.

After applying exclusion criteria (Figure 1), 440 LT recipients were included. Demographic and baseline characteristics of patients excluded from the study did not differ significantly from those included into the study (data not shown). The survival rate at 1 and 5 years in the whole cohort of 706 cases was 83.7% and 70.4%, respectively. The survival rate of the 440 patients included in the study at 1 and 5 years was 77.3% and 69.3%, respectively. The main demographic, clinical, and biochemical characteristics of the study population are summarized in Table 1. Univariate analysis by outcome category of FIB-4 is also reported. Patients who developed advanced liver fibrosis during the follow-up period were older, more frequently transplanted for HCV and diabetics. Hyponatremia and hypoalbuminemia at baseline were more frequent in patients who developed the outcome as compared to those who did not.

### 3.1. Incidence of Advanced Liver Fibrosis

**Figure 2:**
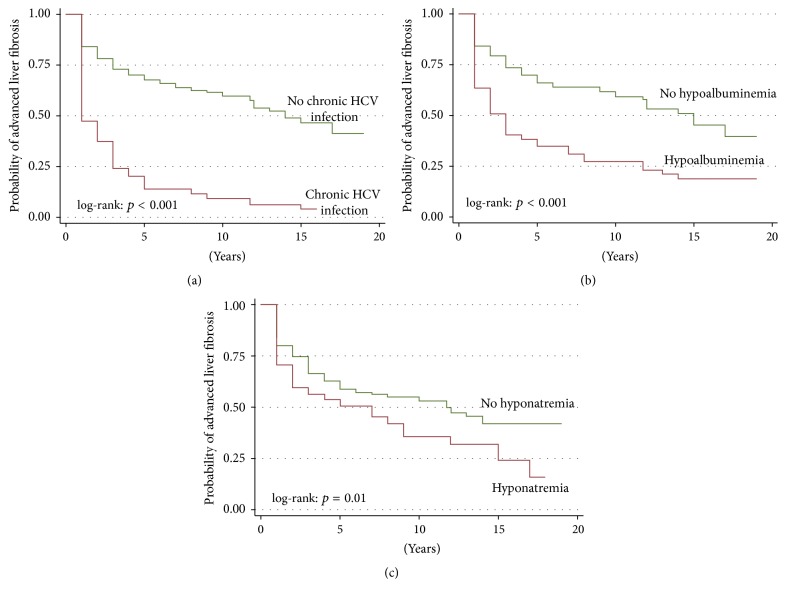
Survival curves of probability of advanced liver fibrosis development by: (a) chronic HCV category; (b) hypoalbuminemia category; (c) hyponatremia category.

Patients were followed up for a median of 4.0 (IQR, 2.0–8.0; range 0.5–20.0) years. Overall, 189 (43%) developed advanced liver fibrosis. Over 2030.5 PY, incidence rate of advanced liver fibrosis was 9.3/100 PY (95% CI, 8.1–10.7). Liver histology was available in 46 out of 189 patients (24.3%), and it confirmed advanced liver fibrosis in 39 cases (84.8%). The 7 discordant cases could be attributed to patients in whom liver biopsy was requested for suspicion of rejection. Overall, the incidence of advanced liver fibrosis was significantly higher in patients with chronic HCV infection (log-rank: *p* < 0.001, Figure 2(a)), those with hypoalbuminemia (log-rank: *p* < 0.001, Figure 2(b)), and patients with hyponatremia (log-rank: *p* = 0.01, Figure 2(c)). We conducted the same analyses with another validated biomarker for liver fibrosis, APRI, and found similar results as for FIB-4 (data not shown).

### 3.2. Predictors of Development of Advanced Liver Fibrosis

**Figure 3:**
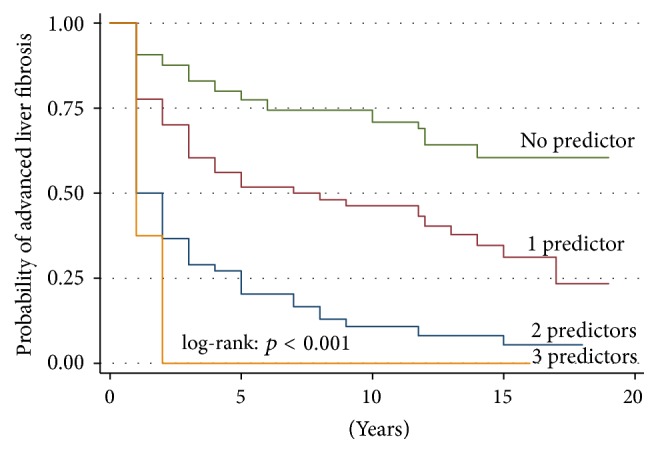
Survival curves of probability of advanced liver fibrosis development according to the number of predictors among chronic HCV infection, hypoalbuminemia, and hyponatremia.

The results of the multivariate Cox regression analysis are shown in Table 2. In this table, the time-dependent and time-independent covariates are listed. After adjustments, development of advanced fibrosis was predicted by chronic HCV infection (adjusted hazard ratio (aHR) = 3.96, 95% CI 2.92–5.36, *p* < 0.001), hypoalbuminemia (aHR = 2.31, 95% CI 1.72–3.09; *p* < 0.001), and hyponatremia (aHR = 1.48, 95% CI 1.09–2.01; *p* = 0.01).  Figure 3 shows development of advanced liver fibrosis during the follow-up period according to the number of predictors among HCV, hypoalbuminemia, and hyponatremia present in the same patient. LT recipients with more than 1 predictor had a higher incidence of advanced liver fibrosis, the highest being for those with coexistence of all 3 predictors (log-rank: *p* < 0.001). The probability of advanced liver fibrosis at 5 and 15 years was 23% and 40% in patients with no predictor, 42% and 69% in those with 1 predictor, 80% and 95% in patients with 2 predictors, and 100% and 100% in those with 3 predictors, respectively. After adjusting for diabetes, cyclosporine therapy, CMV positive recipient, and MELD score, aHR for development of advanced liver fibrosis was 2.38 (95% CI 1.64–3.46, *p* < 0.001) with 1 predictor, 5.42 (95% CI 3.63–8.08; *p* < 0.001) with 2 predictors, and 7.32 (95% CI 3.42–15.69; *p* < 0.001) with 3 predictors. Diabetes and CMV positive recipient were marginally significant as independent predictors of advanced liver fibrosis development.

We also conducted a subgroup analysis assessing predictors of liver fibrosis development in HCV infected recipients and noninfected recipients. In HCV infected recipients, development of advanced fibrosis was predicted by CMV pos recipient (aHR = 1.68, 95% CI 1.05–2.69, *p* = 0.03) and hypoalbuminemia (aHR = 1.88, 95% CI 1.28–2.74; *p* = 0.001), after adjusting for diabetes and cyclosporine treatment. In non-HCV infected recipients, development of advanced fibrosis was predicted by diabetes (aHR = 1.46, 95% CI 1.00–2.12, *p* = 0.04), hyponatremia (aHR = 1.56, 95% CI 1.07–2.27, *p* = 0.02), and hypoalbuminemia (aHR = 2.02, 95% CI 1.40–2.90; *p* < 0.001).

## 4. Discussion

This longitudinal study, based on a large Canadian cohort of well-characterized LT recipients with a long follow-up, confirms that development of advanced liver fibrosis is a frequent event in the overall LT population. Our study identifies chronic HCV infection, hypoalbuminemia and hyponatremia as independent predictors of advanced liver fibrosis following LT. The concurrent presence of all 3 parameters in a single patient is 100% predictive of fibrosis development after LT. The presence of at least one of these 3 predictors indicates the need for greater vigilance in screening for fibrosis after LT. We employed a simple fibrosis biomarker, FIB-4, to characterize incidence and predictors of advanced liver fibrosis. The ready availability of FIB-4 score renders it easy to integrate as a screening tool and for serial monitoring in the busy transplant clinical setting. Such a noninvasive biomarker can be performed over consecutive clinic visits, providing the opportunity to screen for development of advanced fibrosis over time without subjecting the patient to liver biopsies.

The development of hepatic fibrosis after LT portends worse graft survival and need for retransplantation, thus impacting on overall survival [20, 21]. Development of advanced liver fibrosis was frequent in our cohort, with a cumulative incidence of 43% over a median of 4 (IQR, 2–8) years. This figure is in line with other longitudinal studies employing protocol liver biopsies for hepatitis C [22, 23]. We chose to adopt a serum fibrosis biomarker, FIB-4, which can be easily employed by physicians in the clinic setting. Indeed, although liver biopsy is considered the gold standard for liver fibrosis staging, it is invasive, costly, and prone to sampling error. These drawbacks make liver biopsy impractical as a screening tool and for serial monitoring. As such, noninvasive biomarkers are particularly useful for longitudinal assessment of LT recipients. A number of studies conducted in the specific setting of LT have shown that serum fibrosis biomarkers have a diagnostic accuracy similar to pretransplant patients [14, 24, 25]. We chose to employ FIB-4 because it detects advanced liver fibrosis, which is a clinically meaningful endpoint and is fairly validated across various etiologies of chronic liver disease. The 3.25 cut-off value showed a positive predictive value of about 80% [10, 24, 26]. Our findings are corroborated by the fact that liver histology, available in a subgroup of patients, confirmed the diagnosis of advanced liver fibrosis in 84.8% of cases.

Persistent hypoalbuminemia after LT was another independent predictor of advanced liver fibrosis in our study. Albumin is a negative phase reactant, known to decrease with progressive fibrosis [27]. The reduction in functional hepatic parenchyma with advancing fibrosis results in decreased albumin synthesis. Albumin has in fact been incorporated into fibrosis scoring systems such as the NAFLD fibrosis score [28], the SHASTA index [29], and Fibrosis-Cirrhosis Index [30].

Interestingly, an additional parameter predictive of fibrosis was hyponatremia at the time of LT. Hyponatremia is known to adversely affect survival on the transplant waiting list and both short-term outcomes and long-term survival after transplant [31–34]. Even once hyponatremia has resolved after LT, there is still an association with adverse posttransplant outcomes [31]. In fact, 1-year survival was 60.9% among hyponatremic patients as compared to the much higher 78.9% among normonatremic patients. There is a demonstrated survival benefit to LT for patients with a MELD greater than 11 with decreasing serum sodium, supporting implementation of the MELD sodium score [35]. To our knowledge, this is the first study suggesting that the longer-term negative effect of hyponatremia on posttransplant outcomes may be partly due to its association with liver fibrosis progression. Hyponatremia is directly proportional to the degree of vasodilation, which leads to activation of the renin-angiotensin-aldosterone system (RAAS), a major mediator in hepatic fibrogenesis [36–38]. Components of the RAAS, particularly angiotensin II, are expressed in damaged tissue, as is the case in chronic hepatitis [39]. Angiotensin II binds to receptors on myofibroblasts, leading to recruitment of inflammatory cells, extracellular matrix protein release, and prevention of collagen degradation. Angiotensin II also promotes contraction, migration, and proliferation of hepatic stellate cells [36]. Inhibitors of the RAAS have shown significant attenuation of experimental hepatic fibrogenesis in several studies [40–42] and are currently being evaluated in clinical trials [42]. Given the above, our finding of hyponatremia as an independent predictor of fibrosis is physiopathologically consistent. However, it admittedly requires further validation given that MELD sodium did not independently predict fibrosis after LT in our study.

CMV positive recipient status was a marginally significant predictor of development of advanced liver fibrosis by FIB-4 score in our study. This is another known predictor of fibrosis after LT [43], and it has been hypothesized that the fibrotic effect of CMV occurs either by increasing CMV-mediated immunosuppression or by enhancing HCV replication [44]. Diabetes after LT, a condition associated with a systemic inflammatory state, was also marginally predictive of fibrosis after LT. Half of LT recipients with HCV develop posttransplant diabetes, which has been shown to accelerate fibrosis progression and lead to worse graft and patient survival outcomes [45].

The other relevant finding of our study is that progression to advanced liver fibrosis was particularly rapid when two or three predictors among HCV, CMV, hypoalbuminemia, and hyponatremia were present. Our findings suggest that patients with HCV and other concomitant predictors of fibrosis (hypoalbuminemia and/or hyponatremia) should be prioritized for antiviral therapy with the new interferon-free regimens and monitored more closely [46].

We acknowledge certain limitations of our study. First, this was a retrospective study, and as such we were unable to control for potential confounding factors. Second, we estimated the incidence of advanced liver fibrosis based on a surrogate serological biomarker. Indeed, liver biopsies were not performed routinely in our transplant program; rather, they would be done in order to investigate elevated liver tests (thereby assessing for rejection, recurrent hepatitis C, recurrent cirrhosis). However, liver biopsy is impractical as a screening tool and for serial monitoring. Given this constraint, simple serum biomarkers like FIB-4 are easy to implement by clinicians in daily busy clinics and could be used as first-line screening tests, thereby potentially reducing the number of liver biopsies in LT recipients. An additional limitation was the lack of data available on changes in immunosuppression for an individual patient (switching from cyclosporine to tacrolimus or sirolimus) during long-term follow-up.

In conclusion, our longitudinal study shows that advanced liver fibrosis is a frequent occurrence in a large cohort of Canadian LT recipients. Chronic HCV infection, hypoalbuminemia, and hyponatremia are the strongest predictors of advanced fibrosis development. HCV and hypoalbuminemia are known predictors based on previous liver biopsy-based studies. Hyponatremia is an additional intriguing predictor of hepatic fibrosis and requires further prospective study to better elucidate the underlying pathogenetic mechanism. Importantly, our findings suggest that LT recipients with 2 or 3 predictors of advanced fibrosis development among chronic HCV, persistent hypoalbuminemia, and hyponatremia at time of LT should be prioritized for interventions, including antiviral therapy with interferon-free regimens and closer monitoring following LT. This retrospective experience should be independently validated in a prospective manner to further confirm these findings. Nonetheless, given the high concordance we found between FIB-4 and histology, this simple biomarker could be used as first-line screening test and for serial monitoring of LT recipients for liver fibrosis. This would enable earlier recognition of fibrosis and help third-party payers make rational choices regarding prioritization of patients for costly HCV antiviral medications.

## Figures and Tables

**Table 1:** Baseline sociodemographic and clinical characteristics of study population by outcome (FIB-4).

Variable	Total (*n* = 440)	FIB-4 ≤ 3.25 (*n* = 251)	FIB-4 > 3.25 (*n* = 189)	*p* ^*∗*^
Age (years)	55 (10.7)	54.5 (11.2)	57.8 (9.6)	<0.001
Male sex (%)	292 (66.4)	165 (65.7)	127 (67.2)	0.75
Caucasian ethnicity (%)	343 (78)	191 (76.1)	152 (80.4)	0.28
Indication for transplant (%)				
Alcohol	96 (21.8)	59 (23.5)	37 (19.6)	<0.001
HCV	79 (18)	32 (12.7)	47 (24.9)	
NASH	62 (14.1)	34 (13.5)	28 (14.8)	
HBV	34 (7.7)	21 (8.4)	13 (6.9)	
Others	169 (38.4)	105 (41.9)	64 (33.8)	
Obesity (%)	125 (28.4)	70 (27.7)	55 (29)	0.78
Diabetes (%)	93 (21.1)	28 (11.2)	65 (34.4)	<0.001
MELD	22.3 (8.6)	22.1 (8.9)	22.8 (8.2)	0.5
MELD sodium	23.7 (8.2)	23.5 (8.5)	24.1 (7.6)	0.58
Hyponatremia (%)	132 (30)	65 (25.9)	67 (35.4)	0.03
Hypoalbuminemia (%)	150 (34.1)	58 (23)	92 (48.7)	<0.001
CMV pos recipient (%)	296 (67.3)	156 (62.2)	140 (74.1)	0.008
Cholesterol (mmol/L)	4.7 (1.5)	4.6 (1.5)	4.8 (1.4)	0.28
Donor age (years)	44 (17.7)	42.8 (16.7)	51 (15)	0.28
Cold ischemia time (hours)	8.4 (3)	8.3 (3.1)	87 (3)	0.25
Cyclosporine (%)	154 (35)	68 (27.1)	86 (45.5)	<0.001

Results given as mean (standard deviation) or *n* (%). ^*∗*^*T*-test or chi-square test between “FIB-4 ≤ 3.25” and “FIB-4 ≤ 3.25.” BMI, body mass index; ALT, alanine aminotransferase; AST, aspartate aminotransferase; HCV, hepatitis C virus; CMV, cytomegalovirus; HCC, hepatocellular carcinoma; MELD, model for end stage liver disease.

**Table 2:** Multivariate Cox regressions analysis of predictors of development of advanced liver fibrosis (FIB-4 > 3.25).

	Unadjusted HR (95% CI)	*p*	Adjusted HR (95% CI)	*p*
Time-independent baseline covariates				
Hyponatremia at transplant	1.49 (1.1–2.02)	0.01	1.48 (1.09–2.01)	0.01
MELD	1 (0.98–1.02)	0.9	1 (0.99–1.02)	0.63

Time updated covariates				
Diabetes	1.37 (1.01–1.86)	0.04	1.34 (0.98–1.83)	0.06
Hypoalbuminemia	2.55 (1.91–3.4)	<0.001	2.31 (1.72–3.09)	<0.001
CMV pos recipient	1.43 (1.02-2)	0.04	1.36 (0.97–1.892)	0.08
Cyclosporine	0.99 (0.73–1.35)	0.9	1.04 (0.77–1.42)	0.27
Chronic HCV	4.35 (3.22–5.88)	<0.001	3.96 (2.92–5.36)	<0.001

HR, hazard ratio; CI, confidence interval; MELD, model for end-stage liver disease; CMV, cytomegalovirus; HCV, hepatitis C virus.
